# Fast-Velocity Eccentric Cycling Exercise Causes Greater Muscle Damage Than Slow Eccentric Cycling

**DOI:** 10.3389/fphys.2020.596640

**Published:** 2020-12-14

**Authors:** Hisashi Ueda, Yosuke Tsuchiya, Eisuke Ochi

**Affiliations:** ^1^Faculty of Health and Medical Science, Teikyo Heisei University, Tokyo, Japan; ^2^Laboratory of Health and Sports Sciences, Meiji Gakuin University, Kanagawa, Japan; ^3^Faculty of Bioscience and Applied Chemistry, Hosei University, Tokyo, Japan; ^4^Graduate School of Sports and Health Studies, Hosei University, Tokyo, Japan

**Keywords:** lengthening, muscular function, joint flexibility, muscular dysfunction, maximal voluntary concentric contraction torque

## Abstract

This study aims to investigate muscle damage occurring in the early and recovery phases after fast-velocity and slow-velocity eccentric cycling. Eleven untrained men (age, 20.0 ± 1.7 years; height, 171.3 ± 6.8 cm; weight, 61.8 ± 7.7 kg; and %body fat, 13.2 ± 2.9%) performed slow-velocity maximal isokinetic eccentric cycling (slow-velocity; 30°/s) with one leg and fast-velocity (fast-velocity; 210°/s) isokinetic eccentric cycling with the other leg. Changes in maximal voluntary isokinetic concentric contraction (MVCC) torque at velocities of 30 and 210°/s, range of motion (ROM), and muscle soreness were assessed by pressure using a digital muscle stiffness instrument; thigh circumference, muscle echo intensity, and muscle stiffness were assessed before exercise, and immediately after exercise, 1 day, and 4 days after exercise. Comparing with the results obtained for slow-velocity cycling (post: 215.9 ± 32.3 Nm, day 1: 192.9 ± 47.4 Nm, day 4: 184.3 ± 47.2 Nm) and before exercise, MVCC after fast-velocity cycling significantly decreased at immediately (160.4 ± 43.5 Nm), 1 day (143.6 ± 54.1 Nm), and 4 days (150.1 ± 44.5 Nm) after exercise (*p* < 0.05). Significant increase in muscle soreness for vastus lateralis was observed after fast-velocity cycling (41.2 ± 16.9 mm) compared with slow-velocity cycling (23.7 ± 12.2 mm) 4 days after exercise (*p* < 0.05). However, no significant difference in muscle soreness was observed for rectus femoris and vastus medialis at any time points after exercise. In addition, no significant differences were observed in the ROM, thigh circumference, muscle echo intensity, and muscle stiffness. In conclusion, fast-velocity eccentric cycling causes a decrease in muscle strength and an increase in soreness as compared to slow-velocity eccentric cycling.

## Introduction

Continuous resistance exercise is important for maintaining and promoting health as well as for improving athletic performance (American College of Sports Medicine, 2009). Muscle contractions are characterized as isometric, concentric, or eccentric contractions (ECCs). ECCs, in which a contracting muscle is repeatedly lengthened by a greater external force than the muscle force, can induce greater muscle damage in unaccustomed individuals than concentric or isometric contractions (Tsuchiya et al., 2015; Ochi et al., 2016). Muscle damage following ECCs is characterized by a temporal reduction in muscle strength, limitation of range of motion (ROM), development of delayed onset muscle soreness (DOMS), muscle swelling, and increased levels of serum creatine kinase (CK) and myoglobin (Mb; Clarkson and Hubal, 2002; Chen et al., 2009; Tsuchiya et al., 2015; Ochi et al., 2016). Previous studies (Chapman et al., 2006, 2008a) have investigated the effect of ECCs velocities on muscle damage. Shepstone et al. (2005) reported that 30 maximal of fast-velocity ECCs (210°/s) caused greater Z-line disruptions in elbow flexors compared with slow-velocity (20°/s) ECCs. In addition, Chapman et al. (2008a) investigated the changes in indirect markers of muscle damage following 210 ECCs of elbow flexors in slow-velocity (30°/s) and fast-velocity (210°/s) groups; they found that 210 fast-velocity ECCs caused greater reduction in muscle strength, limited ROM, and increased CK levels than 210 slow-velocity ECCs. It is possible that the greater decrements in muscle strength variables post fast-velocity ECCs were due to the greater effects of type II fibers, which are likely to be more susceptible to muscle damage (Chapman et al., 2006). Therefore, fast-velocity ECCs may cause greater muscle damage than slow-velocity ECCs.

Penailillo et al. (2017b) reported that one bout of 30-min ECC cycling exercise induced moderate DOMS and decreased jump performance. More recently, Mavropalias et al. (2020) showed that the muscle function and plasma CK did not differ between high- (20% peak power output for five sets of 1 min) and low-intensity (5% peak power output for five sets of 4 min) ECC cycling under similar mechanical workload and cycling velocity, although they confirmed the difference in DOMS. However, the effect of different velocities on muscle damage caused by ECC cycling exercise has not been investigated. In addition, eccentric cycling targeting the knee flexor muscles may be a beneficial intervention as we could control the speed (i.e., rpm), intensity (i.e., watts), and movement of the muscles without requiring a precise technique (Penailillo et al., 2013).

Herein, we investigate the magnitude of change in indirect muscle damage markers after fast- and slow-velocity ECC cycling. We hypothesize that fast-velocity (210°/s) ECC cycling induces greater muscle damage than slow-velocity (30°/s) ECC cycling.

## Materials and Methods

### Subjects

The sample size was determined using a power analysis (G^*^Power, version 3.0.10) by setting the effect size as 0.25, *α* level as 0.05, and power (1-*β*) as 0.80 for intergroup comparison, which indicated that at least 12 participants were necessary. Thus, 12 young men were recruited [*n* = 11, mean ± standard deviation (SD) age: 20.0 ± 1.7 years; height: 171.3 ± 6.8 cm; body mass: 61.8 ± 7.7 kg; and %body fat: 13.2 ± 2.9%]; however, one participant dropped out. None of the subjects had participated in any regular resistance training in at least 1 year prior to this study. The participants were requested to avoid participation in other clinical trials and interventions, such as hot and cold baths, massage, stretching, strenuous exercise, excessive food, or alcohol consumption, and taking any supplement or medication at least 3 months before and during this trial. All subjects were provided with detailed explanations of the study protocol prior to participation and signed an informed consent form in accordance with the Declaration of Helsinki before being enrolled in this study. Written informed consent was obtained from the individual for the publication of any potentially identifiable images or data included in this article. This study was approved by the Ethics Committee for Human Experiments at Teikyo Heisei University (ID: R01-058-1).

### Experimental Protocols

The subjects randomly performed maximal-effort ECC cycling exercise for 5 min with fast or slow velocity in each leg, such that fast or slow ECC-cycling exercises were performed on the same day by the non-dominant leg of six participants and by the dominant leg of the other five participants, and vice versa. Previous studies have reported that the initial bout of maximal eccentric contractions is responsible for conferring protective effects on the contralateral side (Howatson and van Someren, 2007; Xin et al., 2014; Chen et al., 2016; Tsuchiya et al., 2018). Because this effect occurs when the second bout is performed from 1 day to 4 weeks (Chen et al., 2016), we set the interval between the slow and fast velocities to 15 min. The legs were randomly assigned using a table of random numbers to minimize the intergroup differences in terms of age and body fat. The dependent variables included maximal voluntary isokinetic concentric contraction (MVCC) torque (30 and 210°/s slow and fast velocities, respectively), ROM of the knee joint, muscle soreness assessed using a visual analog scale (VAS), echo intensity, muscle thickness, and shear elastic modulus. These measurements were performed before, immediately after, 1 day, and 4 days after the ECC-cycling exercise. All subjects attended a familiarization session at least 1 week before the exercise where the subjects were briefed on eccentric exercise protocols and MVCC.

### Eccentric Cycling

The velocities of the ECC cycling exercise were either 30°/s (5 rpm; slow velocity) or 210°/s (35 rpm; fast velocity); each velocity was maintained for 5 min using a cycle ergometer (Strength Ergo 240 BK-ERG-003, Mitsubishi Electric Engineering, Tokyo, Japan). The cycling time of 5 min was set based on a previous study (Elmer et al., 2010) and our preliminary experiment. These studies showed that eccentric cycling for 5 min (60 rpm, 360°/s) increased muscle soreness and reduced maximum single-leg concentric cycling power. This ergometer is controlled by a servo motor and can be programmed with various exercise programs using a personal computer. For the testing position, the recumbent position was set at a seat angle, i.e., the angle between the backrest and the seat, was set to 105°, and the pedal shaft was set at 55 cm from ground level (Kato et al., 2011). The subjects were secured with seat belts for safety. The left and right cranks and pedals of the ergometer were all set to the fixed mode, which enabled the subjects to put their feet on the cleated shoes fitted on the pedals and then generate exercise of the dorsal or plantar flexion of the right ankle joint. The positions of the cranks, pedals, and seat were adjusted for enabling the subjects to maintain a comfortable and fixed posture. The subjects were asked to perform all bouts of exercise using either the right or left lower limb (hip and knee joint at 45° of flexion; ankle joint at 0° of plantar/dorsal flexion) and to relax the other lower limb (hip and knee joint at 0° of flexion/extension; relaxed ankle joint) throughout the experiments (Liang et al., 2011). The non-exercising leg was secured to a stabilization platform. The range of motion of the knee joint during cycling ranged from about 20 to 120° (0°, full extension). The peak torque and work performed during cycling were recorded at a 10-Hz sampling rate in a computer connected to the isokinetic dynamometer which is a device similar to the one used for performing eccentric cycling (Strength Ergo 240 BK-ERG-003, Mitsubishi Electric Engineering, Tokyo, Japan).

### Maximal Voluntary Concentric Contraction Torque

**Figure 1:**
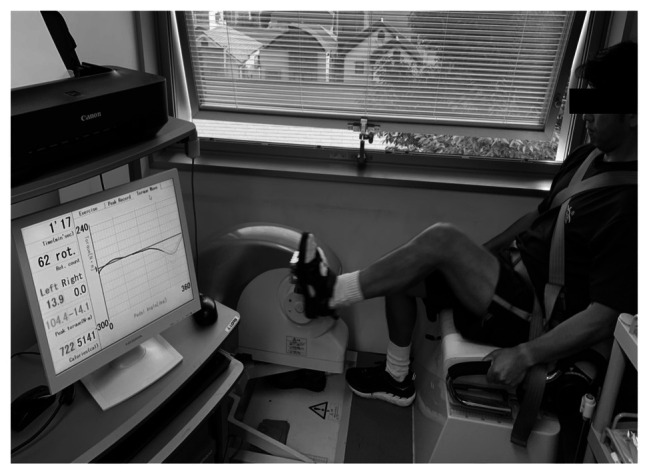
Measurements of eccentric cycling exercise and maximal voluntary isokinetic concentric contraction (MVCC) torque.

Figure 1 shows that the MVCC torque was applied by a cycle ergometer (Strength Ergo 240 BK-ERG-003, Mitsubishi Electric Engineering, Tokyo, Japan). For the measurement of MVCC torque, the subject performed two 5-s MVCCs at 30 and 210°/s with a 60-s resting period between contractions. The peak torque of each velocity was used as the MVCC torque.

### Muscle Soreness

Muscle soreness was assessed using a 10-cm VAS in which 0 indicated “no pain” and 10 indicated the “the worst pain imaginable”; the subject indicated his pain level on this VAS. Muscle soreness was assessed by pressure using a digital muscle stiffness instrument (NEUTONE TDM-NA1, Satou Shouji Inc., Kanagawa, Japan) on vastus lateralis, rectus femoris, and vastus medialis. The pressure was applied perpendicularly to the point on each muscle. All tests were conducted by the same investigator who had practiced to apply the same pressure over time and on different participants.

### Range of Motion

Range of motion was determined as the difference in the joint angles between maximal voluntary flexion and extension of knee joint using a goniometer (Takase Medical, Tokyo, Japan). The flexion was measured when the subject attempted to maximally flex the knee joint of the exercised leg to touch his hip with his heel while keeping the knee joint aligned to the standing leg and supporting the body by placing both hands on the wall, 30 cm from the foot. The extension was measured when the subject attempted to extend the knee joint of the exercised leg as much as possible. ROM was calculated by subtracting the flexion of the knee joint from the extension of the knee joint (Chen et al., 2011, 2013).

### Circumference

When each subject stood with his feet approximately 10 cm apart, with his body weight evenly distributed on both feet, the perimeter distance of the thigh perpendicular to the long axis of the femur at the marked mid-trochanterion-tibiale level was measured (Chen et al., 2011). The measurements were performed thrice for each time point, and the average of the three measurements was used for further analysis.

### Muscle Stiffness, Muscle Thickness, and Echo Intensity

Using ultrasound shear wave elastography, we measured muscle stiffness at vastus lateralis, rectus femoris, and vastus medialis with the probe placed mid-trochanterion-tibiale at the position marked for the circumference measurement. An ultrasonic scanner (Aixplorer version 4.2, Supersonic Imagine, France) was used in shear wave elastography mode with musculoskeletal preset. An electronic linear array probe (SL15-4, Supersonic Imagine France) coated with water soluble transmission gel was placed longitudinally on each muscle head. Muscle shear modulus (*μ*), a measure of normalized muscle stiffness, was calculated using the following equation: *μ* = *ρV*s^2^, where *ρ* is the density of muscle (assumed to be 1,000 kg/m^3^) and *V*s is the velocity of shear wave propagation caused by the focused ultrasound beam from the scanner. A 10-mm square map of the muscle shear modulus with a spatial resolution of 1 × 1 mm was obtained with each ultrasound image. We calculated the average muscle stiffness combining the measurements obtained for vastus lateralis, rectus femoris, and vastus medialis (Lacourpaille et al., 2017). A representative value of the shear modulus for each muscle head was then determined *via* spatial averaging over a 5-mm diameter circle (Ochi et al., 2018). Scanned images of each muscle were transferred to a personal computer and the thicknesses of the vastus lateralis, rectus femoris, and vastus medialis were manually calculated by tracing each muscle using an image analysis software (ImageJ, MD, United States). To measure the echo intensity, the gains and contrast were kept consistent over the experimental period. The transverse images were analyzed in a computer in bitmap (.bmp) format. The average echo intensity for the region of interest (20 × 20 mm) was calculated using ImageJ software that provided a grayscale histogram (0, black; 100, white) for the region, as described in a previous study (Tsuchiya et al., 2019b).

### Statistical Analyses

All analyses were performed using the SPSS software version 25.0 (IBM Corp., Armonk, NY, United States). Values are expressed as means ± SD. The peak torque and work performed during eccentric cycling and the baseline data for all outcomes at slow and fast velocities were compared using the paired *t* test. Time courses of MVCC torque, ROM, circumference, shear elastic modulus, muscle thickness, and echo intensity of values were calculated based on relative changes from the baseline. MVCC torque, ROM, muscle soreness, echo intensity, muscle thickness, and shear elastic modulus were compared between the slow-velocity and fast-velocity groups *via* two-way repeated-measure analysis of variance (ANOVA). When a significant main effect or interaction was detected, Bonferroni’s correction was performed for the *post-hoc* testing. MVCC torque, ROM, circumference, shear elastic modulus, muscle thickness, and echo intensity were also adjusted for the pre-value of the MVCC torque *via* analysis of covariance (ANCOVA). The partial eta squared (*η*^2^) values were calculated to demonstrate the effect sizes. The intraclass correlation coefficient (ICC) was used for examining the test-retest reliability for all the measured variables. A *p* < 0.05 was considered statistically significant.

## Results

### Peak Torque and Work Performed During ECC Cycling

**Figure 2:**
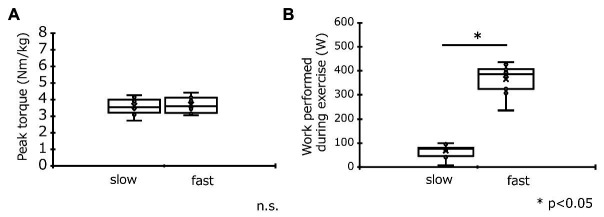
Comparisons (mean ± SD) of peak torque **(A)** and work performed **(B)** during eccentric cycling in the slow-velocity (slow) and fast-velocity (fast) groups. ^*^Denotes a significant (*p* < 0.05) difference between slow-velocity and fast-velocity group results.

No significant difference was observed in peak torque during ECC cycling between the slow-velocity (3.6 ± 0.5 Nm/kg) and fast-velocity (3.6 ± 0.5 Nm/kg) groups (Figure 2A). The work performed during ECC cycling was significantly lower at slow velocity (68.3 ± 26.6 W) than fast velocity (365.7 ± 60.6 W; *p* < 0.05; see Figure 2B).

### Maximal Voluntary Concentric Contraction Torque

**Figure 3:**
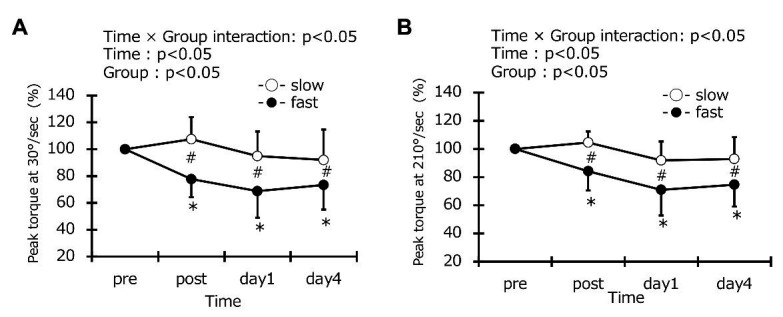
Changes (mean ± SD) in peak isokinetic torque at 30°/s **(A)** and 210°/s **(B)** before (pre), immediately after (post), 1 day, and 4 days after exercise in the slow-velocity (slow) and fast-velocity (fast) groups. ^*^Denotes a significant (*p* < 0.05) difference from the pre-value in the fast-velocity group. ^#^Denotes a significant (*p* < 0.05) difference between slow-velocity and fast-velocity group results.

Maximal voluntary concentric contraction torque at the baseline was the same between the two groups (slow-velocity group: 206.4 ± 42.7 Nm, fast-velocity: 205.4 ± 35.9 Nm). A significant interaction effect was found in MVCC torque at 30°/s (*p* < 0.05, *η*^2^ = 0.189). Moreover, MVCC torque at 30°/s in the fast-velocity group was significantly lower than slow-velocity group immediately after exercise (post exercise in the slow-velocity group: 106.6 ± 15.9%, post exercise in the fast-velocity group: 77.7 ± 13.5%; *p* < 0.05) and remained low for up to 4 days after exercise (day 1, slow-velocity group: 94.6 ± 19.2%, fast-velocity group: 68.8 ± 20.0%; day 4, slow-velocity group: 91.5 ± 23.0%, fast-velocity group: 73.4 ± 18.4%; p < 0.05; Figure 3A). Compared with the pre-exercise value, MVCC torque at 30°/s in the fast-velocity group significantly decreased immediately after exercise and remained decreased up to 4 days after exercise (*p* < 0.05). However, MVCC torque in the slow-velocity group did not change at all time points after exercise. These results are similar to those obtained at 210°/s (slow-velocity group, post: 104.5 ± 7.9%, day 1: 91.9 ± 13.4%, day 4: 92.8 ± 15.6%; fast-velocity group, post: 84.2 ± 13.6%, day 1: 71.0 ± 18.2%, day 4: 74.6 ± 15.5%; *η*^2^ = 0.174; *p* < 0.05; Figure 3B). The results of ANCOVA also detected x times interaction in the groups (*p* < 0.05). ICC values for MVCC at 30 and 210°/s were 0.95 and 0.97, respectively.

### Muscle Soreness

**Figure 4:**
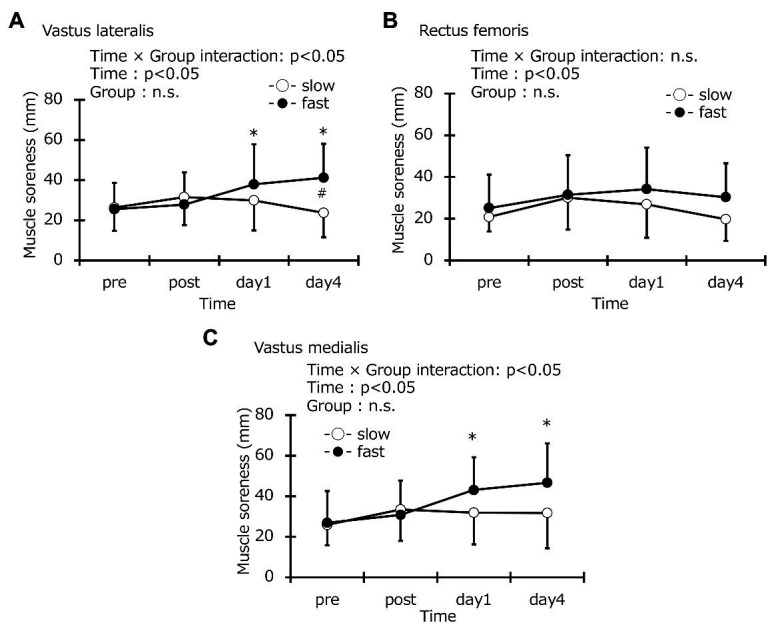
Changes (mean ± SD) in muscle soreness recorded using a visual analog scale for the vastus lateralis **(A)**, rectus femoris **(B)**, and vastus medialis **(C)** before (pre), immediately after (post), 1 day, and 4 days after exercise in the slow-velocity (slow) and fast-velocity (fast) groups. ^*^Denotes a significant (*p* < 0.05) difference from the pre-value in the fast-velocity group. ^#^Denotes a significant (*p* < 0.05) difference between slow-velocity and fast-velocity group results.

Muscle soreness in vastus lateralis at the baseline was the same in the two groups (slow-velocity group: 26.3 ± 11.5 mm, fast-velocity group: 25.5 ± 13.1 mm). Similarly, muscle soreness in vastus medial and rectus femoris at baseline were also the same in the two groups (vastus lateralis: slow-velocity group: 20.8 ± 6.9 mm, fast-velocity group: 25.1 ± 16.0 mm; rectus femoris: slow-velocity group: 25.9 ± 10.1 mm, fast-velocity group: 26.9 ± 15.7 mm). A significant interaction effect was found in the vastus lateralis (*p* < 0.05, *η*^2^ = 0.267; Figure 4). However, no significant interaction effect was found in the vastus medial and rectus femoris using the VAS (vastus medial: *η*^2^ = 0.147, rectus femoris: *η*^2^ = 0.051; Figure 4). A significant increase in muscle soreness of the vastus lateralis, rectus femoris, and vastus medialis was observed in the fast-velocity group at 1 and 4 days after exercise. However, the muscle soreness in all muscles of the slow-velocity group did not change at all after exercise. A significant increase in muscle soreness was observed only in the vastus lateralis between the two groups 4 days after exercise (slow-velocity group: 23.7 ± 11.5 mm, fast-velocity group: 41.2 ± 16.9 mm; *p* < 0.05). The results of ANCOVA did not affect the interpretation of the main findings. The ICC values for muscle soreness on vastus lateralis, rectus femoris, and vastus medialis were 0.86, 0.94, and 0.95, respectively.

### Range of Motion of Knee Joint and Circumference

**Figure 5:**
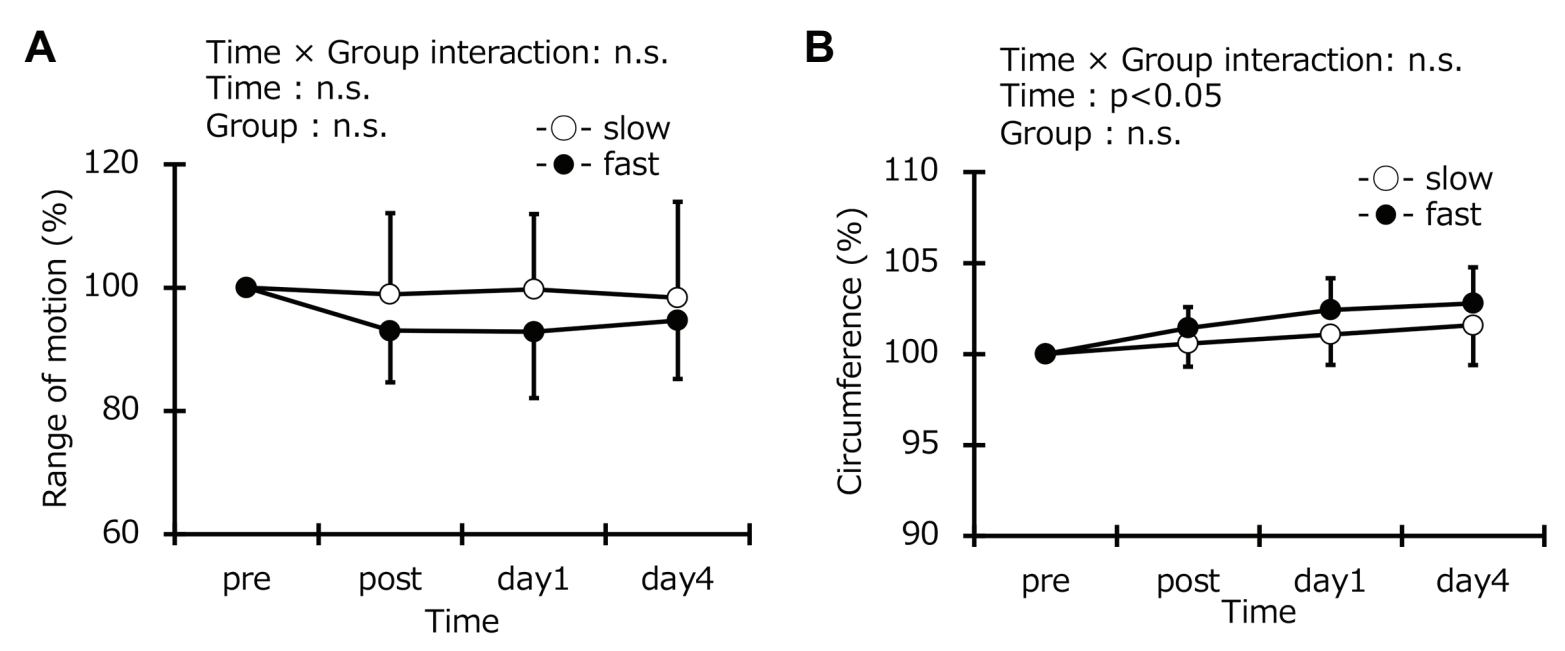
Changes (mean ± SD) in range of motion **(A)** and thigh circumference **(B)** before (pre), immediately after (post), 1 day, and 4 days after exercise in the slow-velocity (slow) and fast-velocity (fast) groups.

Range of motion at baseline was the same in the two groups (slow-velocity group: 120.5° ± 17.2°, fast-velocity group: 119.4° ± 8.8°). No significant interaction was found in ROM (*η*^2^ = 0.053; Figure 5A). No significant change in the ROM from the pre-exercise values for the slow-velocity and the fast-velocity groups were observed at any time point after exercise (Figure 4A). The thigh circumference measurements at baseline were the same in the two groups (slow-velocity group: 49.4 ± 3.5 cm, fast-velocity: 48.9 ± 3.2 cm) and no significant interaction was found (*η*^2^ = 0.041). Circumference also did not show any change between the slow-velocity and fast-velocity groups (Figure 5B). The ANCOVA for ROM and circumference also did not have an interaction effect. The ICC value for ROM was 0.89.

### Muscle Stiffness, Thickness, and Echo Intensity

**Figure 6:**
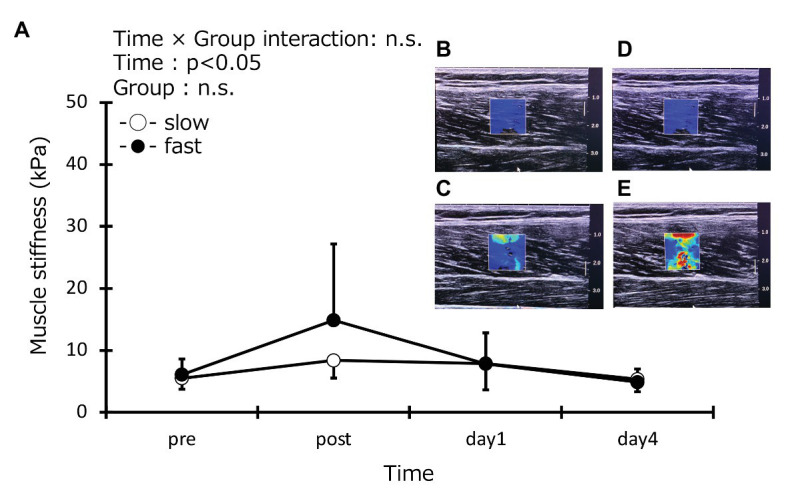
Muscle stiffness of knee extensors (vastus lateralis, rectus femoris, and vastus medialis) before (pre), immediately after (post), 1 day, and 4 days after eccentric contractions in the slow-velocity (slow) and fast-velocity (fast) groups **(A)**. Representative images of muscle stiffness in the slow-velocity (slow) group before **(B)** and immediately after **(C)** and in the fast-velocity (fast) group before **(D)** and immediately after **(E)** exercise.

**Figure 7:**
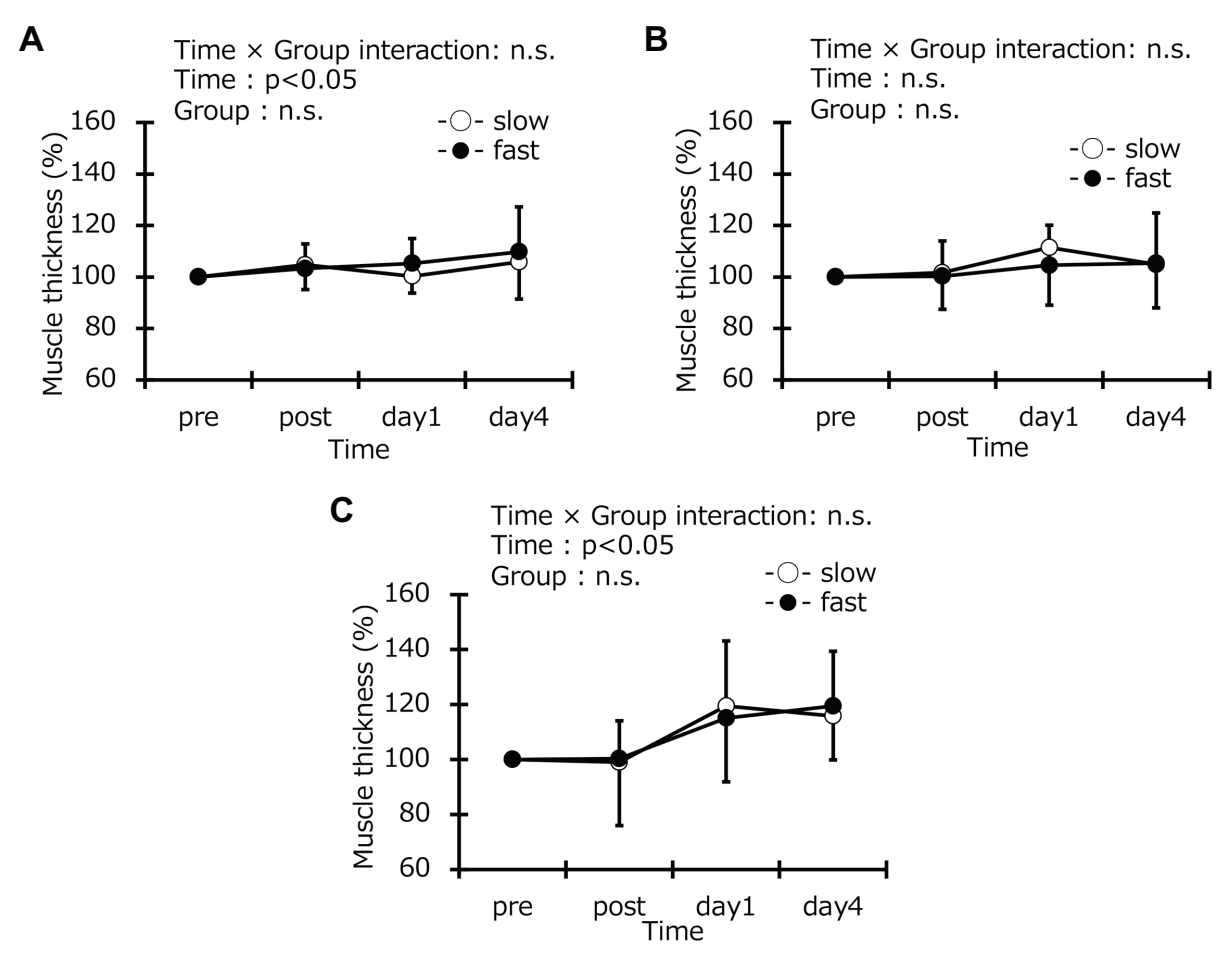
Muscle thickness of vastus lateralis **(A)**, rectus femoris **(B)**, and vastus medialis **(C)** before (pre), immediately after (post), 1 day, and 4 days after exercise in the slow-velocity (slow) and fast-velocity (fast) groups.

**Figure 8:**
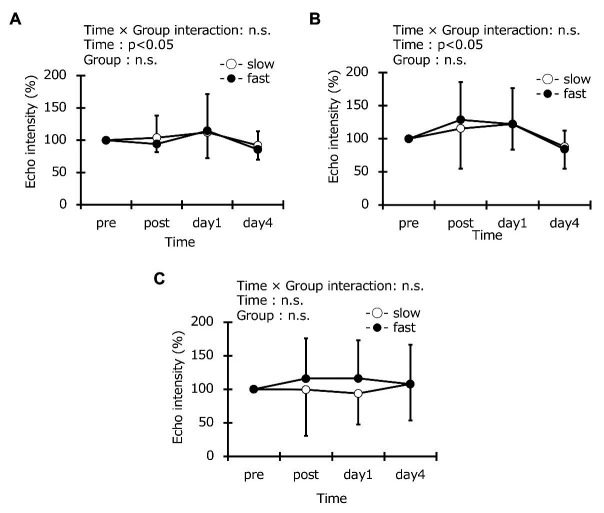
Echo intensity of vastus lateralis **(A)**, rectus femoris **(B)**, and vastus medialis **(C)** before (pre), immediately after (post), 1 day, and 4 days after eccentric contractions in the slow-velocity (slow) and fast-velocity (fast) groups. n.s. indicates that no significant interaction effect was observed.

Muscle stiffness at baseline was the same in the two groups (slow-velocity group: 5.5 ± 1.7 kPa, fast-velocity group: 6.1 ± 2.5 kPa; Figure 6). In addition, the muscle thickness in the vastus lateralis at baseline was the same in the two groups (slow-velocity group: 2.1 ± 0.3 cm, fast-velocity group: 2.0 ± 0.3 cm; Figure 7). Similarly, vastus lateralis and rectus femoris at baseline also were the same in the two groups (vastus lateralis: slow-velocity group: 2.5 ± 0.3 cm, fast-velocity group: 2.5 ± 0.3 cm; rectus femoris: slow-velocity group: 2.2 ± 0.4 cm, fast-velocity group: 2.3 ± 0.4 cm). Echo intensity in the vastus lateralis at baseline was the same in the two groups (slow-velocity group: 32.2 ± 9.6 AU, fast-velocity group: 36.2 ± 9.3 AU; Figure 8). Similarly, the vastus lateralis and rectus femoris at baseline were also the same in the two groups (vastus lateralis: slow-velocity group: 26.8 ± 10.1 AU, fast-velocity group: 26.1 ± 8.3 AU; rectus femoris: slow-velocity group: 23.3 ± 6.6 mm, fast-velocity group: 23.0 ± 6.6 mm). No significant interaction was found in muscle stiffness (*η*^2^ = 0.099; Figure 7). No significant difference was observed in muscle stiffness in knee extensors between slow- and fast-velocity groups (Figure 6). In addition, no significant interaction was found in muscle thickness and echo intensity (muscle thickness: vastus lateralis: *η*^2^ = 0.034, vastus medial: *η*^2^ = 0.014, and rectus femoris: *η*^2^ = 0.021; echo intensity: vastus lateralis: *η*^2^ = 0.011, vastus medial: *η*^2^ = 0.020, and rectus femoris: *η*^2^ = 0.013). No significant changes in the muscle thickness and the echo intensity were observed at any time point among all muscles. In addition, the ANCOVA for muscle stiffness, thickness, and echo intensity did not detect any interaction effects. The ICC values were 0.82 for muscle stiffness; 0.96, 0.97, and 0.88 for muscle thickness in the vastus lateralis, vastus medial, and rectus femoris, respectively; and 0.96, 0.95, and 0.98 for echo intensity in the vastus lateralis, vastus medial, and rectus femoris, respectively.

## Discussion

This study compared the levels of muscle damage after transient fast- and slow-velocity ECC cycling. We found that fast-velocity ECC cycling is associated with significantly greater decreases in the MVCC torque at both 30 and 210°/s than slow-velocity ECC cycling. Furthermore, fast-velocity ECC cycling is associated with significantly higher DOMS in the vastus lateralis than slow-velocity ECC cycling. Otherwise, flexibility of joints and swelling, stiffness, and echo intensity of muscles did not differ significantly between the fast- and slow-velocity groups. These results indicate that faster pedaling induces a greater decrease in muscle strength and DOMS than slower pedaling in the ECC cycling. This result substantiates our hypothesis.

The peak torque during ECC cycling did not differ significantly between the slow- and fast-velocity groups (Figure 2A). The peak torque was larger in our study as compared to a previous study (Penailillo et al., 2015) because of the difference in maximal effort used (Penailillo et al. study: 73.5 ± 17.3 Nm vs. our study: slow-velocity: 220.5 ± 8.7 Nm, fast-velocity: 224.7 ± 3.4 Nm). We suggest that this difference was due to the difference in the intensity of the concentric workload (Penailillo et al. study: 65% vs. our study: 100%). Moreover, fast velocity was found to be associated with a significantly higher value of work performed than slow velocity (Figure 2B). This result is similar to that of the previous studies (Chapman et al., 2006, 2008a). Hence, although different exercise contractions were used in our study, the results of our study agree with those of the previous studies (Chapman et al., 2006, 2008a).

Herein, we evaluated MVCC torque values before and immediately after, day 1 and 4 after exercise under two contraction velocities of 30 and 210°/s and found that values obtained at fast velocity were significantly lower than those obtained at slow velocity under both measurement conditions (Figures 3A,B); this supports the results of previous studies (Chapman et al., 2006, 2008a). Morgan and Allen (1999) proposed that post-ECCs muscle weakness is due to disturbance in excitation-contraction coupling or the damage of muscle cell membrane involved in muscle contraction and degeneration of structural proteins. In addition, Shepstone et al. (2005) have demonstrated that a fast ECC exercise protocol causes more disruption of Z-lines than a slow exercise protocol. Although morphological changes at a muscle fiber or myofibril level were not directly observed in present study, it is reasonable that the greater decrease in muscle strength in the fast-velocity group can be attributed to micro-damage to muscle fiber.

In this study, muscle soreness in the vastus lateralis 4 days after fast-velocity ECC cycling was significantly higher than that after slow-velocity ECC cycling (Figure 4A). However, no differences between the two groups were observed for the vastus medialis and rectus femoris (Figures 4B,C). Differing results among these muscles are attributable to the activation of muscle fibers. A previous study compared electromyographic activities (EMG) in the quadriceps during ECCs and CON cycling exercises, showing that the EMG amplitude during exercise was greater in the following order: vastus lateralis (17.3 ± 8.7%), vastus medialis (15.4 ± 4.4%), and rectus femoris (13.7 ± 7.3%; Penailillo et al., 2017a). Although a different exercise mode was used, another study compared EMG amplitudes in the vastus lateralis, vastus medialis, and rectus femoris muscles during lateral step-up, forward lunge, and monopodial squat exercises, reporting that mean EMG during all exercises normalized to maximal voluntary isometric contraction in the vastus lateralis were significantly higher than those in the rectus femoris (Muyor et al., 2020). The second bout of eccentric cycling (separated from the first bout by 2 weeks) in the vastus lateralis EMG amplitude was lower than the first bout, and muscle soreness developed only after the first bout (Penailillo et al., 2015). The vastus lateralis was deactivated or relaxed in the second bout which could be associated with less muscle soreness. Based on the results of this study and previous studies, we suggest that, among muscles in the quadriceps, the vastus lateralis more active and, therefore, damaged more after fast ECC cycling. However, the relationship between muscle activity and muscle damage after ECC cycling exercise should be investigated in a more detailed manner.

Range of motion after ECC cycling did not significantly differ between the two groups (Figure 5A). However, previous studies have reported that ROM was reduced in a fast elbow flexion ECC model (Chapman et al., 2006, 2008a). The ROM reduction has been mechanistically attributed to the inflammatory reaction in the myofibrils after ECCs and is related to increased muscle stiffness and swelling (Chleboun et al., 1998). In contrast, a previous study comparing 30 and 210 contractions showed no decrease in ROM after 30 contractions at slow or fast velocity (Chapman et al., 2008a). The 5-min exercise may have no effects on ROM regardless of contraction velocity in the ECC cycling exercise used in this study. Further research is required to study the effects of ECCs over long durations on ROM. No significant differences in the thigh circumference were found between the two groups (Figure 5B). This result is similar to the findings of a previous study involving elbow flexion ECCs (Chapman et al., 2008a). The limit of detection may not have been sufficient to detect the effects of the different contraction velocities on muscle swelling because a measuring tape was used to measure the thigh circumference similar to a previous study (Chen et al., 2013). Similarly, no differences in muscle thickness were observed in any of the muscles measured (Figure 7). Our previous study has shown that cross-sectional areas (CSA) of the upper arms, measured using the magnetic resonance imaging (MRI) method, increased after ECCs of elbow flexors (Tsuchiya et al., 2019a). These data suggest that the MRI evaluation of CSA is necessary for future studies. The time effect was significant in the vastus lateralis and vastus medialis, although no differences were observed between the two groups (Figure 8) in this study. Edema was seen to occur in the vastus lateralis and vastus medialis for the cycling model used in this study because the increased echo intensity is presumed to reflect muscle edema (Nosaka and Clarkson, 1996). No previous studies have investigated the effects of different contraction velocities on the echo intensity. Future studies may be needed for further clarification.

Changes in muscle stiffness after ECCs reflect disturbance of calcium homeostasis rapidly progressing after exercise-induced destruction of myofibrils (Lacourpaille et al., 2014, 2017). Lacourpaille et al. (2017) compared muscle stiffness (considering vastus lateralis, rectus femoris, and vastus medialis together) at three relaxed knee joint angles (30, 90, and 110°) after ECCs (60°/s) in knee extensors under a low load (15 × 5 sets) and a high load (30 × 5 sets) conditions. They have reported no differences between pre- and post-exercise stiffness at a knee joint angle of 30° in both groups, significant increases 30 min after exercise at a knee joint angle of 90° only under the high load condition, and at 110° under both conditions. They have also reported that the increase in muscle stiffness under the high load condition was significantly more than that under the low load condition (Lacourpaille et al., 2017). No significant difference in muscle stiffness before and after cycling and between groups was found in this study. Muscle stiffness in the fast-velocity group showed a tendency to be higher than in the slow-velocity group in this study, although the interaction effect was not significant. We speculate that the fast-velocity cycling utilizes the fast-twitch fibers because a previous study suggests that the reasons for increased muscle stiffness at a high load condition could utilize more fast-twitch fiber than slow-velocity cycling (Lacourpaille et al., 2017). Therefore, further studies focusing on this are needed.

This study has several limitations. The work performed was different between the two trials as shown in Figure 2. Similarly, Chapman et al. (2008a) also showed that the total work performed in the elbow flexors with fast velocity (210°/s) was greater than slow velocity (30°/s), and the fast velocity caused greater muscle damage than slow velocity. Another study reported that total work, change in work, torque developed, and change in torque developed during maximal ECCs of elbow flexors did not correlate with the magnitude of changes in common indirect markers of muscle damage (Chapman et al., 2008b). However, the effect of work performed cannot be excluded in this study. Hence, further studies are needed that compare fast and slow velocities at the same work output. In addition, exercise time was set at 5 min in this study based on a previous study (Elmer et al., 2010). Although the previous studies set exercise time to 10–60 min, they examined under sub-maximal exercise. Elmer et al. (2010) showed that maximal eccentric cycling for 5 min (60 rpm, 360°/s) caused an increase in muscle soreness and a reduction in maximum single-leg concentric cycling power. However, it is possible that slow-velocity ECCs cycling duration was too short to cause muscle damage. Hence, it is necessary to conduct a study on the effect of cycling with slow velocity for longer duration on muscle damage.

## Conclusion

In conclusion, we demonstrated that the fast-velocity ECC cycling exercise induced a decrease in muscle strength and an increase in muscle soreness compared with the slow-velocity ECC cycling exercise. In contrast, no effects of the ECC cycling velocity on joint flexibility or muscle swelling were observed. Future studies should address effects of the exercise duration on muscle damage and muscle adaptation after repeated bouts of cycling exercises. The findings of this study provide useful information, evidencing that velocity is a factor that determines levels of muscle damage and soreness associated with ECC cycling.

## Data Availability Statement

The raw data supporting the conclusions of this article will be made available by the authors, without undue reservation.

## Ethics Statement

The studies involving human participants were reviewed and approved by the Ethics Committee for Human Experiments at Teikyo Heisei University (ID: R01-058-1). The patients/participants provided their written informed consent to participate in this study.

## Author Contributions

EO designed the study. HU and YT measured, collected and analyzed the data and wrote the main parts of the manuscript. HU, YT, and EO reviewed the manuscript. All authors contributed to the article and approved the submitted version.

### Conflict of Interest

The authors declare that the research was conducted in the absence of any commercial or financial relationships that could be construed as a potential conflict of interest.
